# Mechanistic Insights Into Co-Administration of Allosteric and Orthosteric Drugs to Overcome Drug-Resistance in T315I BCR-ABL1

**DOI:** 10.3389/fphar.2022.862504

**Published:** 2022-03-18

**Authors:** Hao Zhang, Mingsheng Zhu, Mingzi Li, Duan Ni, Yuanhao Wang, Liping Deng, Kui Du, Shaoyong Lu, Hui Shi, Chen Cai

**Affiliations:** ^1^ School of Chemistry and Chemical Engineering, Shaoxing University, Shaoxing, China; ^2^ Department of Plastic and Reconstructive Surgery, Shanghai Ninth People’s Hospital, Shanghai Jiao Tong University, School of Medicine, Shanghai, China; ^3^ Department of Anesthesiology, Huashan Hospital Affiliated to Fudan University, Shanghai, China; ^4^ Medicinal Chemistry and Bioinformatics Center, Shanghai Jiao Tong University, School of Medicine, Shanghai, China; ^5^ Department of VIP Clinic, Changhai Hospital, Navy Medical University, Shanghai, China; ^6^ Department of Respiratory and Critical Care Medicine, Changhai Hospital, Navy Medical University, Shanghai, China

**Keywords:** chronic myelogenous leukemia (CML), BCR-ABL1, tyrosine kinase inhibitors (TKIs), nilotinib, drug resistance, ABL001, allosteric drug, combinatory treatment

## Abstract

Chronic myeloid leukemia (CML) is a myeloproliferative neoplasm, driven by the BCR-ABL1 fusion oncoprotein. The discovery of orthosteric BCR-ABL1 tyrosine kinase inhibitors (TKIs) targeting its active ATP-binding pocket, such as first-generation Imatinib and second-generation Nilotinib (NIL), has profoundly revolutionized the therapeutic landscape of CML. However, currently targeted therapeutics still face considerable challenges with the inevitable emergence of drug-resistant mutations within BCR-ABL1. One of the most common resistant mutations in BCR-ABL1 is the T315I gatekeeper mutation, which confers resistance to most current TKIs in use. To resolve such conundrum, co-administration of orthosteric TKIs and allosteric drugs offers a novel paradigm to tackle drug resistance. Remarkably, previous studies have confirmed that the dual targeting BCR-ABL1 utilizing orthosteric TKI NIL and allosteric inhibitor ABL001 resulted in eradication of the CML xenograft tumors, exhibiting promising therapeutic potential. Previous studies have demonstrated the cooperated mechanism of two drugs. However, the conformational landscapes of synergistic effects remain unclear, hampering future efforts in optimizations and improvements. Hence, extensive large-scale molecular dynamics (MD) simulations of wide type (WT), WT-NIL, T315I, T315I-NIL, T315I-ABL001 and T315I-ABL001-NIL systems were carried out in an attempt to address such question. Simulation data revealed that the dynamic landscape of NIL-bound BCR-ABL1 was significantly reshaped upon ABL001 binding, as it shifted from an active conformation towards an inactive conformation. The community network of allosteric signaling was analyzed to elucidate the atomistic overview of allosteric regulation within BCR-ABL1. Moreover, binding free energy analysis unveiled that the affinity of NIL to BCR-ABL1 increased by the induction of ABL001, which led to its favorable binding and the release of drug resistance. The findings uncovered the in-depth structural mechanisms underpinning dual-targeting towards T315I BCR-ABL1 to overcome its drug resistance and will offer guidance for the rational design of next generations of BCR-ABL1 modulators and future combinatory therapeutic regimens.

## Introduction

Targeted drug therapy strikes specifically at defined carcinogenic targets representing a therapeutic breakthrough in human cancer treatments (Bozic et al., 2013). Kinase, one of the largest gene families, is a class of critical drug targets (Manning et al., 2002; Qiu et al., 2021a; Wang et al., 2021), and kinase inhibitors have revolutionized cancer therapeutics. Traditionally, most kinases inhibitors target the ATP-binding site, also known as orthosteric site. Prolonged administration of inhibitors often leads to inevitable emergence of resistant mutations, which subsequently results in diminished therapeutic efficacy (Nussinov et al., 2017, 2021; Agnello et al., 2019), posing a huge challenge to drug development and threatening global public health (Holohan et al., 2013). Compared to orthosteric ligands bounding to conserved orthosteric sites, allosteric modulators bind to structurally diverse allosteric sites (Lu et al., 2021) and yield pivotal advantages in terms of high specificity and selectivity in the paradigm of precision medicine (Guarnera and Berezovsky, 2016; Lu et al., 2019c; Lu and Zhang, 2019). In addition, allosteric drugs could act together with orthosteric inhibitors to exert synergistic and allosteric effects on a protein, potentially restoring or even boosting the efficacy of orthosteric inhibitors (Ma et al., 2016; Huang et al., 2021). Hence, co-administration of allosteric and orthosteric inhibitors offers a revolutionary strategy to conquer the notorious problem of drug resistance (Al-Lazikani et al., 2012; Nussinov et al., 2017; Ni et al., 2020a; Zhang et al., 2020).

The most recent successful example of combining allosteric and orthosteric drugs to circumvent drug resistance is the treatment of chronic myeloid leukemia (CML) by targeting Breakpoint Cluster Region-Abelson1 (BCR-ABL1) kinase (Heisterkamp et al., 1985). CML is driven by the reciprocal translocation between chromosomes 9 and 22, leading to the fusion of the Breakpoint Cluster Region (BCR) and Abelson1 (ABL1) genes on the Philadelphia chromosome (Ph) (Apperley, 2015). The expression of BCR-ABL1 which has a constitutively active ABL1 kinase domain leads to aberrant activation of numerous signaling pathways finally resulting in the dysregulated differentiation, growth, and survival of leukemic cells (Melo, 1996). In recent years, the prognosis of CML patients has been improved due to the promotion of tyrosine kinase inhibitors (TKIs), including imatinib, dasatinib, nilotinib (NIL) and ponatinib, inhibiting the kinase activity of BCR-ABL1 by targeting its adenosine triphosphate (ATP) -binding site (Jabbour and Kantarjian, 2016). More than 80% of patients treated with TKI had improved survival rates of more than 10 years (Kalmanti et al., 2015). However, some patients suffer from loss of response to TKIs, usually associated with drug resistance generated by BCR-ABL1 kinase mutations that impede drug binding (Pophali and Patnaik, 2016). In particular, the T315I gatekeeper mutation with a frequency up to 30% in BCR-ABL1 (Chahardouli et al., 2013), is resistant to first- and second-generation TKIs except ponatinib whose dosing is limited by adverse events (Quintás-Cardama et al., 2007; Miller et al., 2014). To tackle the notorious problem of drug resistance, researchers have proposed the possibility of combinatorial treatments with allosteric and orthosteric drugs to against drug resistance.

Here we characterize Asciminib (ABL001), the first allosteric BCR-ABL1 inhibitor successfully entering phase III clinical trial and marketed (Deeks, 2022), which synergistically inhibits the BCR-ABL1 mutant with orthosteric drugs. Remarkably, previous studies have confirmed that the dual targeting towards BCR-ABL1 utilizing ABL001 and NIL resulted in complete disease control and eradication of the CML xenograft tumours without recurrence after discontinuation of treatment (Wylie et al., 2017), highlighting the great potential of the combinatory therapeutics of orthosteric and allosteric molecules. Previous studies have described the mechanism of BCR-ABL allosteric drugs and how they working conjointly with orthosteric drugs (Adrián et al., 2006; Zhang et al., 2010). Nevertheless, the detailed mechanism in conformational ensembles of their cooperative inhibition remains unclear.

Therefore, we applied extensive large-scale molecular dynamics (MD) simulations of wide type (WT), WT-NIL, T315I, T315I-NIL, T315I-ABL001 and T315I-ABL001-NIL systems to unravel the detailed molecular mechanistic of dual-targeting therapeutics to overcome drug resistance. Among these complexes, T315I, and T315-NIL are considered as resistant systems whose enzymatic activities were not successfully inhibited; whereas WT-NIL, T315I-ABL001, and T315I-ABL001-NIL are considered as sensitive systems, where the kinases within were successfully inactivated. Dissection of conformation landscapes of these systems found that the dynamic conformation of the ternary complex is the most stable. ABL001 could shift NIL-bound active BCR-ABL1 to inactive conformation by modulating the conformation of key structural domains utilizing Markov state model (MSM) analysis. The binding free energy analysis showed that the affinity of NIL to BCR-ABL1 was strengthened upon ABL001 binding, thus exerting concerted effects on improving the overall therapeutic efficacy. The community network of allosteric signaling was described, to gain an atomistic view of allosteric regulation within BCR-ABL1. Moreover, the allosteric crosstalk between the allosteric site and ATP-binding pocket was investigated utilizing energetic dynamics computations.

Collectively, the findings uncovered the in-depth structural mechanisms underpinning dual-targeting towards BCR-ABL1. This will help offer guidance for the rational design of future generations of BCR-ABL1 modulators and provide novel insights into the regulation of receptor tyrosine kinase (Nussinov et al., 2014). The cooperative targeting of orthosteric and allosteric inhibitors to address drug resistance provides proof of exemplifying for clinical optimization of co-administration therapy in the future.

## Results

### Overview of the Ternary Complex Structure

**FIGURE 1:**
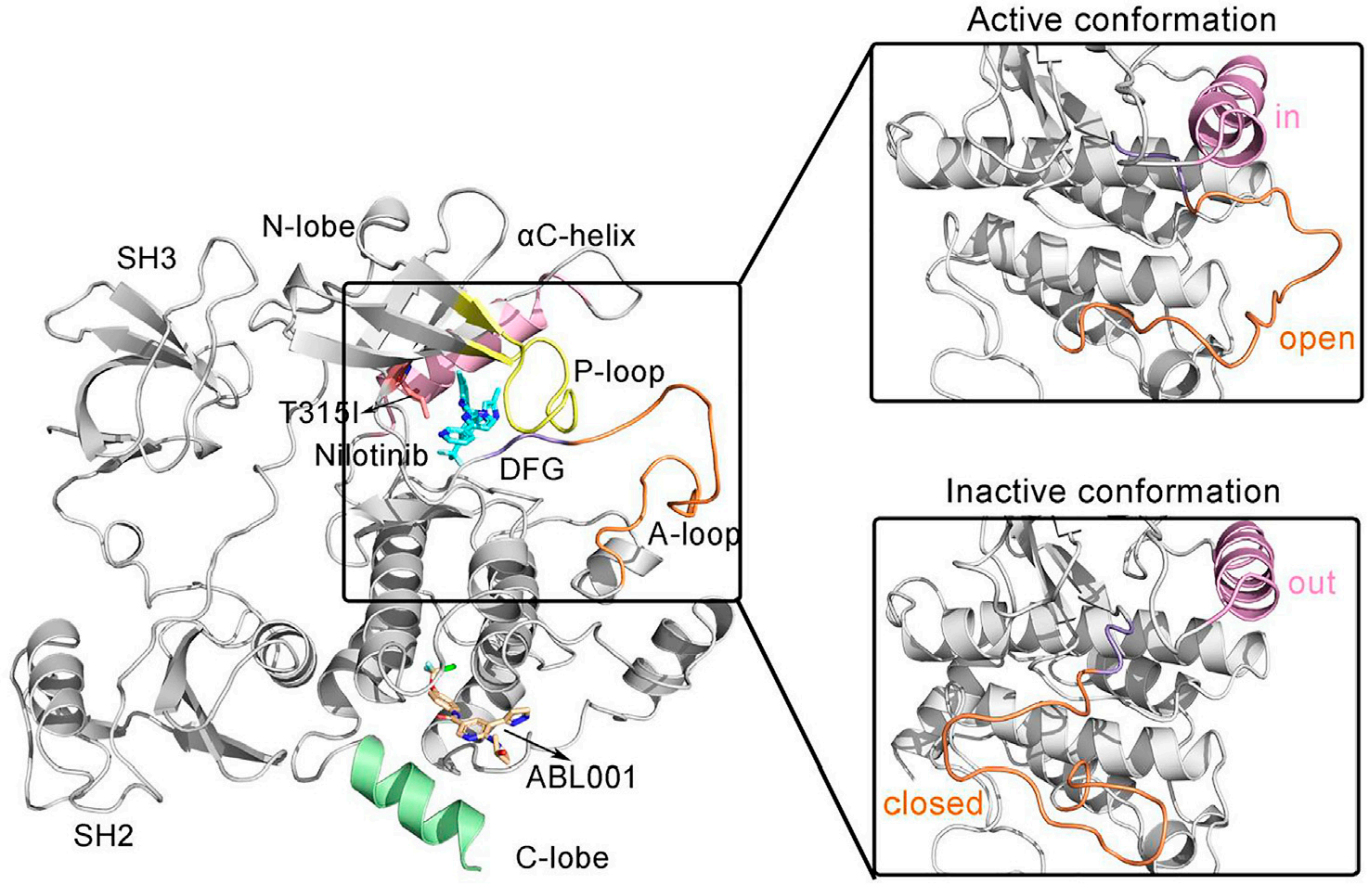
The overall structure of the allosteric drug ABL001 (off-white) bound to the myristoyl pocket and the second generation TKI NIL (cyan) bound to the ATP-pocket in a ternary complex with BCR-ABL1 fusion protein (PDB ID: 5mo4). The backbone of BCR-ABL1 structure is represented in gray. Key structural elements are highlighted in orange (A-loop), yellow (P-loop), pink (αC-helix), and purple (DFG motif), respectively.

The structural domain of BCR-ABL1 protein is organized similarly to Src family kinases, with contiguous Src homolog 2 (SH2) and SH3 domains, an SH2/kinase linker, and a bilobal kinase domain (Figure 1) (Panjarian et al., 2013).

SH3 and SH2 domains are among the most common modular protein-protein interaction domains in human proteins (Sherbenou et al., 2010). Structurally, the SH3 domain consists of two short antiparallel β-sheets forming a barrel-shaped structure, and the SH2 domain comprises a central antiparallel β-sheet flanked by α-helices. Deletion or mutations of the SH3 and SH2 structural domains results in upregulation of ABL1 kinase activity, suggesting that the SH3 and SH2 structural domains together inhibit kinase activation (Mayer and Baltimore, 1994).

The core catalytic domain of kinase adopts a bilobed architecture, consisting of a small N-terminal lobe (N-lobe) followed by a large C-terminal lobe (C-lobe), which is in charge of catalyzing the transfer of γ-phosphate from ATP to tyrosine residues of substrate proteins. The N-lobe consists of β1-β5 sheets and an important αC-helix. A glycine-rich loop (or Wolker loop) connects the β1 and β2 strands, also known as the P-loop that is critical for binding ATP. The P-loop is highly flexible, so it can be interspersed between the β- and γ-phosphates of the substrate ATP to facilitate phosphoryl transfer reactions. Whereas the C-lobe consists mainly of α-helices. Within the C-lobe, the activation loop (A-loop), the most flexible part of BCR-ABL1, is a principal regulatory structure for modulating kinase activity (Schindler et al., 2000; Sonti et al., 2018). A conserved “DFG” motif (D381-F382-G383) motif implicated in ATP binding is located at the N-terminus of A-loop (Lovera et al., 2012). In the active conformation of Abl1 kinase, the A-loop is in an “open” or “extended” conformation. In this conformation, the body of the A loop does not block the C-lobe, enabling the C-lobe to be available for binding the substrate. The D381 within the DFG conserved motif is oriented toward the ATP binding pocket, also known as DFG-in active conformation, and its carboxylic acid functional group binds the Mg coordinated to ATP in active kinases. While in the “closed” form, the A-loop could block substrate binding to the C-lobe. The Abl kinase domain switches from an active from to an inactive form, resulting in a conformational change at the start of the A loop. This flips the orientation of the DFG motif by 180°, termed DFG-out conformation (Shan et al., 2009). With the Asp side chain is flipped away from the ATP binding site, Mg coordination (with the Mg-ATP complex) is prevented. Additional conformational changes upon the ABL1 activity transition include the rotation orthogonal to main axis of αC-helix (Fajer et al., 2017). In the active ABL1 kinase, the αC helix is rotated inward, also known as the “in” position, allowing a salt bridge to form between E286 and K271 in the β-III sheet lining the ATP binding pocket. This salt bridge between two highly conserved residues is considered to be critical for a catalytically competent kinase active site. In the inactive state, E286 along the αC-helix points away from the binding pocket, adopting the “out” conformation. E286 whereas interacts with Arg386 in the A-loop and the salt bridge breaks (Paul et al., 2020). Furthermore, there is a “myristate binding pocket” located in the C-lobe. The myristoyl group covalently attached to the N-terminal end of ABL1 prior to fusion with BCR and induced an assembly inactive state (Nagar et al., 2003). However, this natural autoregulation mechanism disappears after the BCR fragment replaces the N-terminal cap region of ABL1 containing the myristoylation site, resulting in structural activation of BCR-ABL1.

The N-lobe and C-lobe of the kinase are connected by a hinge domain, which contains several conserved residues that provide the catalytic machinery and make up an essential part of the ATP binding pocket, also participating in substrate binding. The catalytic pocket situates at the interface between the N-lobe and C-lobe where the substrate binds. TKIs currently approved for the treatment of CML were designed to target the catalytic ATP binding site of BCR-ABL1, whose efficacy tends to be compromised due to resistance mutations. Especially, there is a “gatekeeper” residue Thr315 within this pocket involved in ATP binding that plays a critical role in stabilizing the active conformation of BCR-ABL1 (Liu et al., 1998). The T315I gatekeeper mutation always leads to recalcitrant resistance to TKIs, with a frequency up to 30% in BCR-ABL1 (Gorre et al., 2001).

NIL, a second-generation TKI, was computationally designed to overcome BCR-ABL1’s resistance to the first-generation TKI imatinib (Kantarjian et al., 2011). Although NIL is effective in suppressing most mutations within BCR-ABL1, it still failed to counteract the T315I gatekeeper mutation. However, recent research has shown that the notorious T315I resistance could be tamed by the combination of ABL001 and NIL without recurrence after the cessation of treatment. In contrast to catalytic-site ABL1 kinase inhibitors, ABL001 was developed using fragment-based NMR screening X-ray for the myristate pocket of ABL1 that is normally occupied by the myristoylated N-terminal of ABL1—a motif that serves as an allosteric negative regulatory element lost upon fusion of ABL1 to BCR (Schoepfer et al., 2018). Structural analysis showed that ABL001 forms hydrophobic interactions mainly with BCR-ABL1, and induces an inactive kinase conformation. To explore the mechanism underlying cooperatively double targeting of ABL001 and NIL towards BCR-ABL1, MD simulations were carried out for six systems, WT, WT-NIL, T315I, T315I-NIL, T315I-ABL001, and T315I-ABL001-NIL, in an attempt to pursue deeper insight into the drug combinations to overcome T135I resistance.

### Allosteric Drug Enhanced System Stability

We calculated the Cα atoms root-mean-square deviation (RMSD) of each system versus initial structures to interrogate the dynamic conformational alterations during simulations. RMSD data indicated that all six systems began to reach equilibrium after 600 ns of simulations (Supplementary Figure S1A), and our further analyses focused on the trajectories under equilibrium states. The RMSD values for the six systems were 3.35 ± 0.43(WT), 2.90 ± 0.6(WT-NIL), 3.13 ± 0.33(T315I), 3.20 ± 0.45(T315I-NIL), 3.10 ± 0.48(T315I-ABL001) and 2.67 ± 0.39 Å (T315I-ABL001-NIL), respectively. They were not significantly different among the systems, implying that the single mutation T315I did not affect the overall protein stability. The RMSD value of the T35I-ABL001-NIL system was the lowest, suggesting this system might exhibit the most stable conformational dynamics.

**FIGURE 2:**
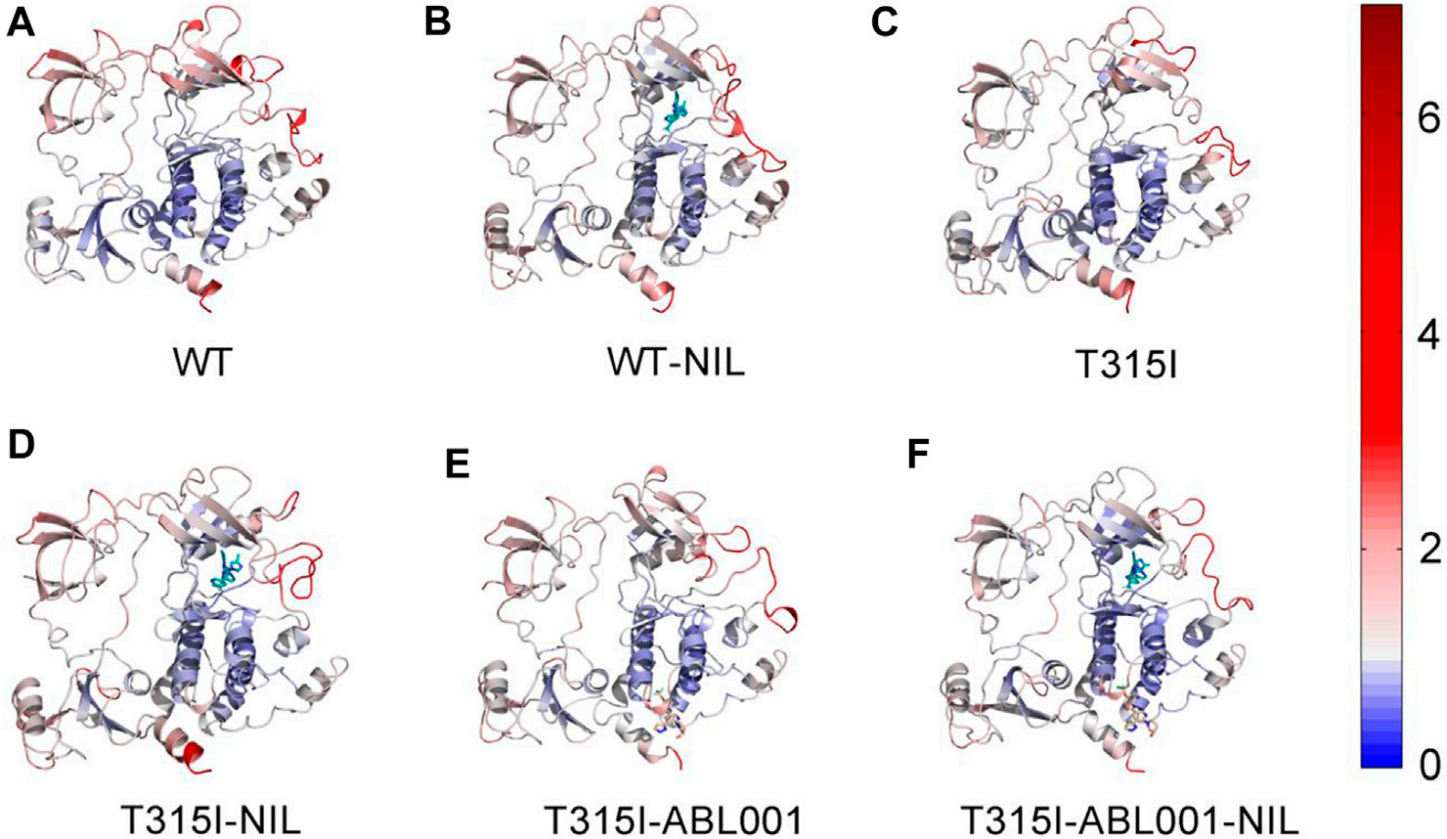
The Cα RMSFs of WT **(A)**, WT-NIL **(B)**, T315I **(C)**, T315I-NIL **(D)**, T315I-ABL001 **(E)** and T315I-ABL001-NIL **(F)** systems along 3,000 ns MD simulations were projected onto the structure of BCR-ABL1.

To quantify the local conformation dynamics of BCR-ABL1, the atomic root-mean-square fluctuations (RMSFs) of Cα atoms around its original position were calculated for each residue (Supplementary Figure S1B), which was projected onto the structure of ABL1 in each system for visualization (Figure 2). The RMSF values for the six systems were 1.30 ± 0.71(WT), 1.35 ± 0.80(WT-NIL), 1.28 ± 0.72(T315I), 1.30 ± 0.63(T315I-NIL), 1.26 ± 0.61(T315I-ABL001) and 1.17 ± 0.64 Å (T315I-ABL001-NIL), respectively. Analysis of the RMSF profiles of the six systems showed that the T35I-ABL001-NIL system typically displayed a lower RMSF, suggesting that this system was relatively more stable. Notably, the functional domains such as αC-helix and A-loop, which played critical roles in the binding of TKIs, displayed relatively higher RMSFs in each system, demonstrating their elasticity and critical implication in TKI drugging. We thus mainly focused on these domains in following analysis.

**FIGURE 3:**
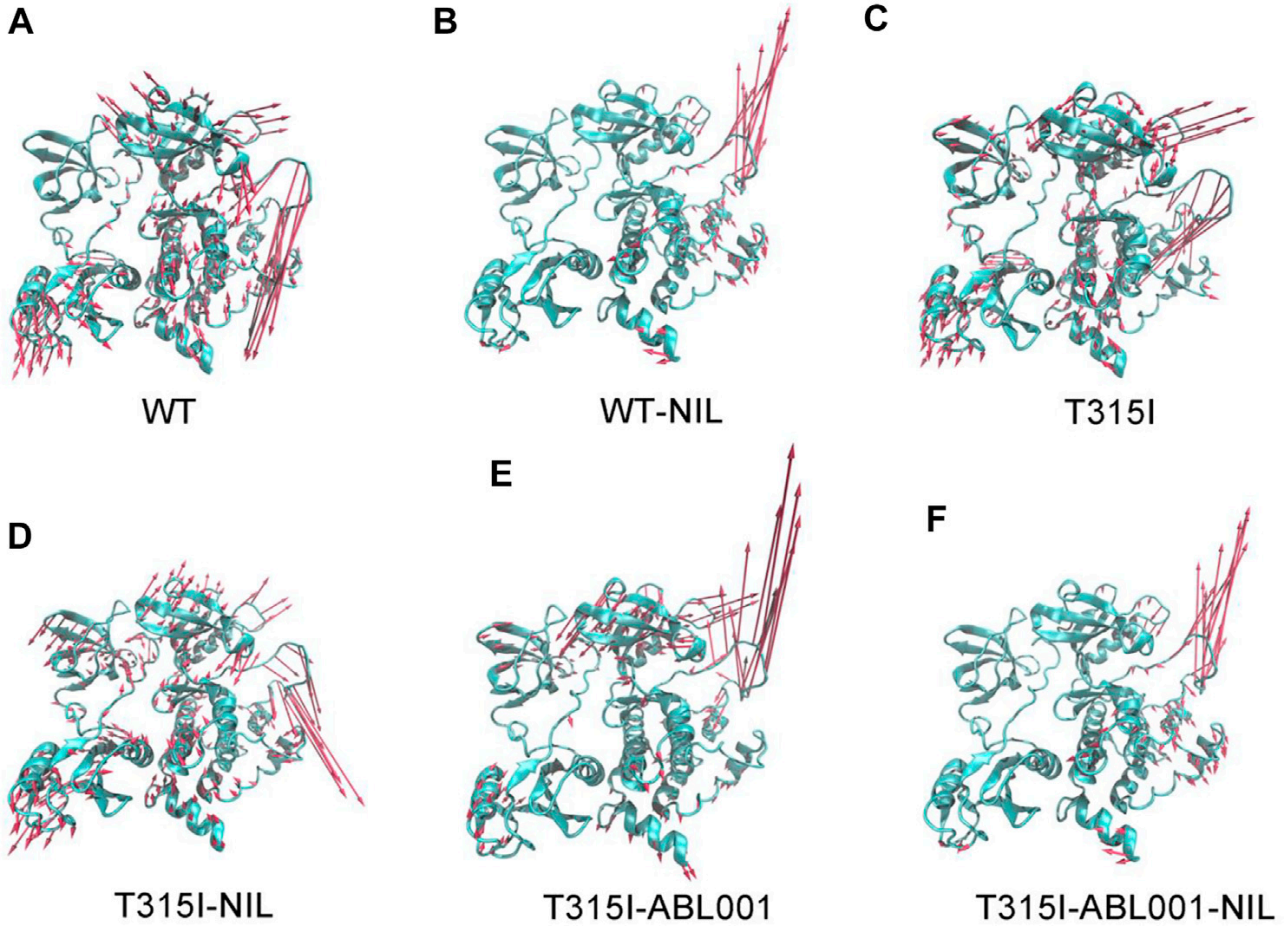
Comparison of principal mode of motion along PC1 in WT **(A)**, WT-NIL **(B)**, T315I **(C)**, T315I-NIL **(D)**, T315I-ABL001 **(E)** and T315I-ABL001-NIL **(F)** systems. Red arrows depict the directions of protein motions, while the length of the arrows represents the magnitude of the movements.

Principal component analysis (PCA) was next conducted to identify the predominant overall conformational transitions of BCR-ABL1 (Amadei et al., 1993). Porcupine diagrams were constructed where PC1 was projected onto initiation structure of each system to graphically visualize the dominant motions of different regions in BCR-ABL1 throughout the simulation (Figure 3).

The principal dynamic motions of BCR-ABL1 system mainly resided on its A loop and αC-helix, which is in accordance with the RMSF data. Particularly, systems exhibited the most prominent differences in the motion vectors of A-loop. We observed that NIL relieved the downward movement of the A-loop in the WT system and conversely induced an upward motion. It thus locked the kinase in an inactive conformation. In contrast, in the T315I-NIL system, the A-loop remained a downward opening orientation possibly due to the hindrance from the resistant T315I mutation towards NIL. Furthermore, in the T315I-ABL001 and T315I-ABL001-NIL systems, ABL001 inhibited kinase activity by allosteric regulation during which the A-loop, readout of kinase activity, exhibited elevat motion.

Overall, in the WT, T315I and T315I-NIL systems where BCR-ABL1 was in active state, the global movement trends of the protein were large. While the overall kinase movement tendency was smaller and the global structures were more stabilized in the WT-NIL, T315I-ABL001 and T315I-ABL001-NIL systems, which were in accordance with their successful inhibition by different drugs respectively, especially in the dual-targeting ternary system.

### Drug Combination Limited Correlated Motions Between Domains

**FIGURE 4:**
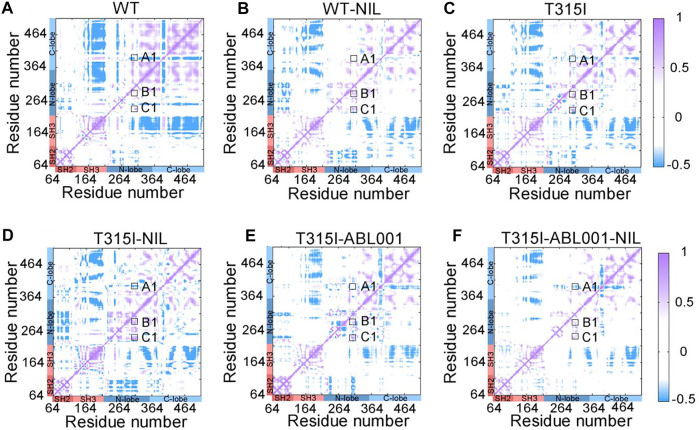
DCCM plots of  WT **(A)**, WT-NIL **(B)**, T315I **(C),** T315I-NIL **(D)**, T315I-ABL001 **(E)**, and T315I-ABL001-NIL **(F)** systems. Positive areas (purple) represent correlated motion, while negative areas (blue) stand for anti-correlated motion. Correlation motions with absolute values less than 0.3 are ignored and displayed in white.

To explore the intra-chain correlation within all systems, residue interactions in each system were quantified through dynamic cross-correlation matrices (DCCMs) (Figure 4). The dynamic correlated motions of the WT-NIL system were markedly reduced compared with the WT system, indicating that NIL might stabilize the residual interactions within BCR-ABL1, conferring its inhibitory activity. However, compared with the T315I system, the T315I-NIL system exhibited a slight elevation of correlated motions in the T315I-NIL system, which may be related to the inability of NIL to suppress T315I mutation. In contrast, the T315I-ABL001 system exhibit a lower value of DCCM globally. Overall, in the T315I-ABL001-NIL system, the dynamic correlated motion amplitude was the lowest (Figure 4F), indicating that the residue interactions within this system were the most stable, which was consistent with the RMSF and PCA analysis.

In particular, we observed that DCCM was weakened in A-loop (A1) and enhanced in the αC-helix (B1) and P-loop (C1) in the T315I system, compared with WT system, implying that the T315I mutation was anti-correlated with A-loop whereas positively associated with the movements of αC-helix and P-loop. In the T315I system, the binding of NIL failed to suppress the mutation, and A1, B1, C1 all became larger, enhancing the coupling between the mutated residue T315I and A-loop, αC-helix and P-loop. While A1, B1, and C1 were slightly reduced in the T315I-ABL001 complex compared with T315I system, implying ABL001 led to weakened coupling. Furthermore, in the co-administered T315I-ABL001-NIL system, A1, B1, and C1 were the weakest, indicating the weakest coupling between T315I and important structural domains mentioned above. The outcome is consistent with the clinical fact that the combination of TKI and ABL001 can successfully inhibit T315I BCR-ABL1, while TKI alone failed to inhibit T315I.

### Co-Administration of NIL and ABL001 Reprogrammed Structural Community and Allosteric Signal Network Within BCR-ABL1

**FIGURE 5:**
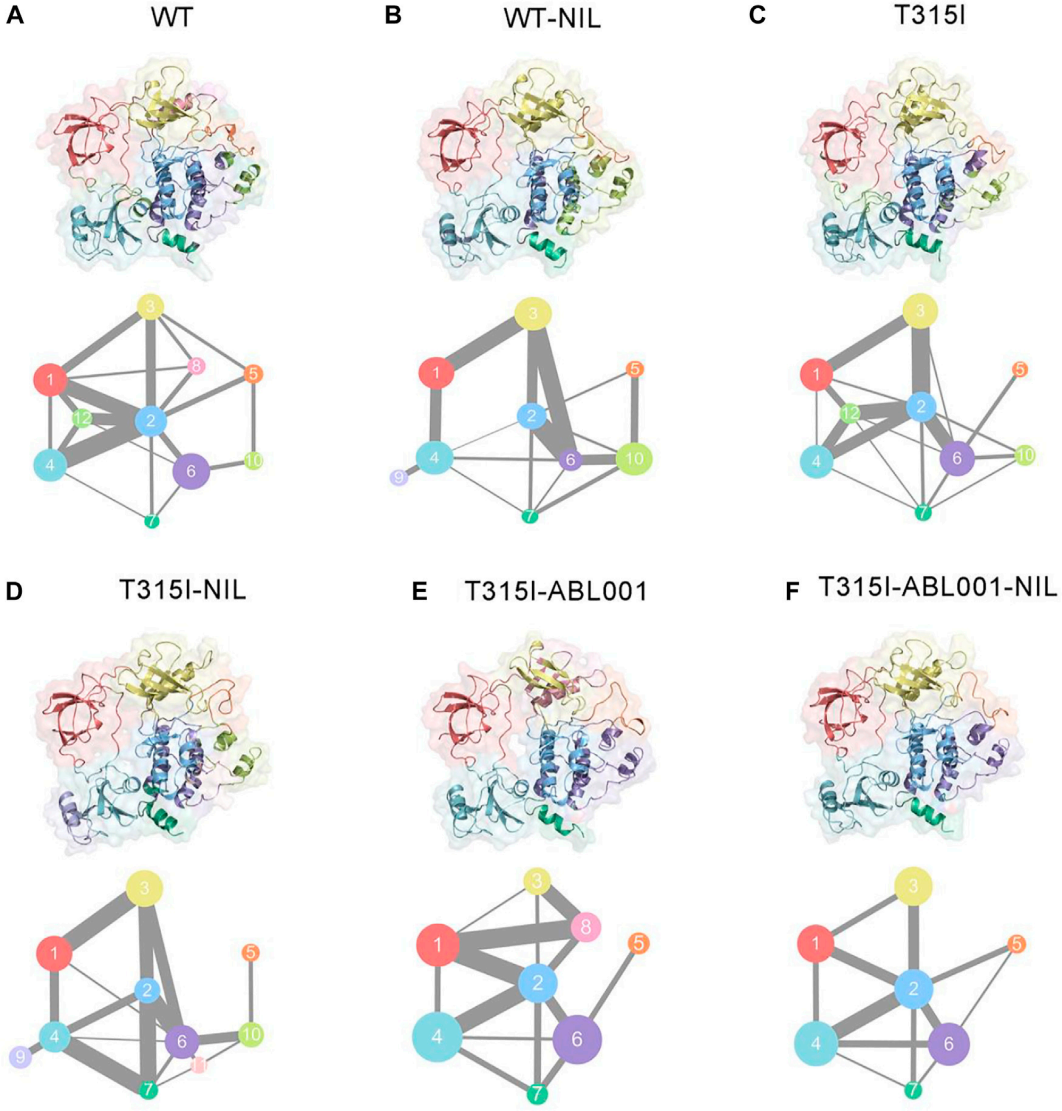
Colored community networks of WT **(A)**, WT-NIL **(B)**, T315I **(C),** T315I-NIL **(D)**, T315I-ABL001 **(E)**, and T315I-ABL001-NIL **(F)**. Each sphere stands for an individual community with an area proportional to the number of residues it contains. While the lines connecting different spheres visualize the inter-community connections, and the thickness of these lines is proportional to the corresponding edge connectivity.

We next explored the propagation pathways of the allosteric signal through community analysis based on the Girvan-Newman algorithm, and quantitatively estimated the variational coupling among the communities. Throughout the trajectory, residues within a cut-off distance of 4.5 Å for at least 75% of simulation time were classified in the same community, which were considered as a synergistic functional unit within the overall structure. The visualized community network graphs clearly depicted the paths and the corresponding intensity of allosteric crosstalk, allowing visual comparison of the allosteric network within BCR-ABL1 among different systems (Figure 5).

In the WT system, community one was composed of SH3, while the majority of SH2 formed community 4, except for the connection between SH3 and SH2 that constituted community 12. Community three consisted of the majority of the C-lobe, including the P-loop. The αC-helix constituted community 8, and the rest of the C-lobe, A-loop, made up community 5. The N-lobe contained communities 2, 6, 7, and 10, where communities 2, 6, 7 enclosed the allosteric cavity of ABL001. Compared with WT (Figure 5A), upon NIL loading, community eight of was incorporated into community 2, indicating that the inhibitor strengthened the association within BCR-ABL1 N-lobe and might thereby capture the kinase in an inactive state (Figure 5B). When NIL was loaded into the T315I system (Figure 5D), community 11, representing the myristoyl pocket within the C-lobe, was independent from community six and had diminished signal linkage to surrounding residues as NIL failed to inhibit T315I BCR-ABL1 kinase activity. And the connection between community one and community three becomes weaker in T315I-ABL001 system compared with T315I system, suggesting that ABL001 could induce a reduction in the perturbation of SH3 and N-lobe (Figure 5E). In the T315I-NIL-ABL001 system, distinct alterations were observed for the topologic characteristics and the inter-community communications within the BCR-ABL1 allosteric network compared with T315I-NIL system. Communities 10 and 11 that are close to the allosteric site are incorporated into community 6, indicating an enhanced signal transduction near the allosteric site, possibly explaining the allosteric effect in distant regions. The incorporation of community nine into community four within SH2 implies that the allosteric effect promoted the internal signal of the SH2 region. In addition, the connection between Community two and Community five was strengthened upon ABL001 binding, indicating an enhanced allosteric signal flow between them. This implied that some less important connections were quenched upon inhibitor binding, but specific signaling pathways were promoted to transmit the effects of the ligands.

Overall, there were 10 communities and 15 pathways in the T315I-NIL complex, 9 communities and 14 pathways in the T315I-AB001, and 8 communities and 11 pathways in the T315I-ABL001-NIL complex (Figure 5F). The reduction in the number of communities and the overall complexity of community connectivity following drug binding implied that the co-binding of inhibitors significantly remodeled the topology of communities, possibly accounting for their inhibition.

### Co-Binding of NIL and ABL001 Shifted BCR-ABL1 Towards Inactive State

**FIGURE 6:**
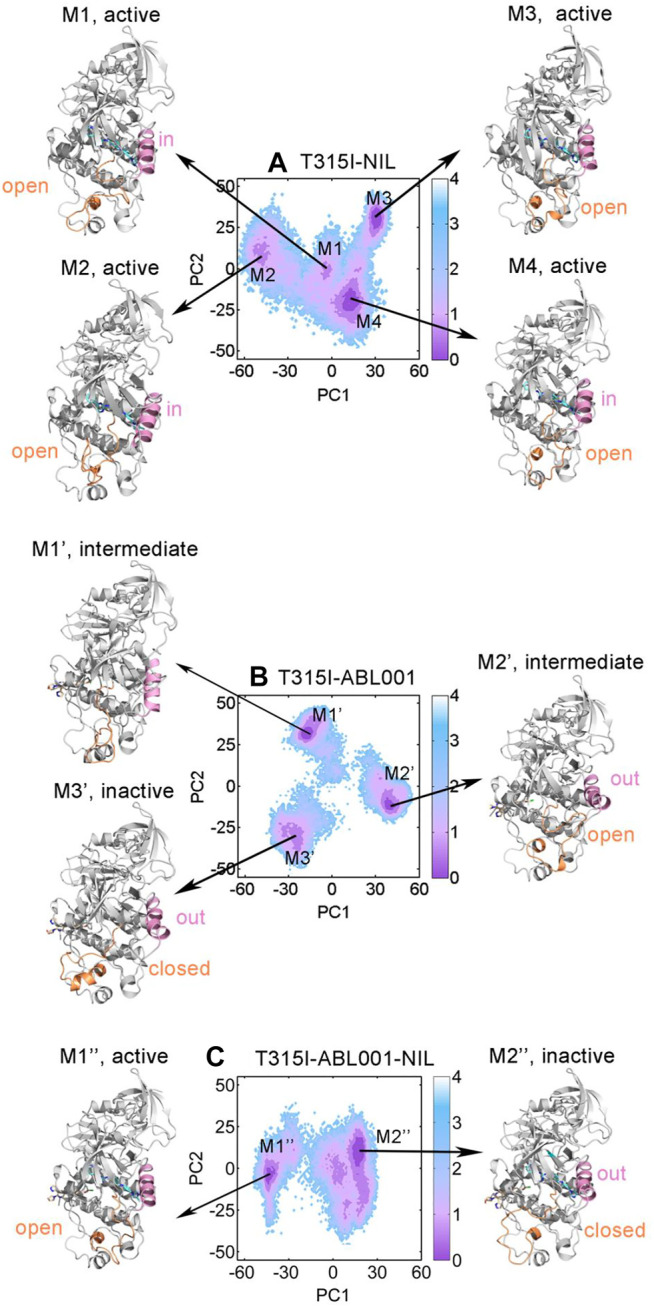
Conformational landscapes of T315I-NIL **(A)**, T315I-ABL001 **(B)** and T315I-ABL001-NIL **(C)** were generated using PCA. Structural comparison of dominant intermediate state of T315I-NIL (M1, M2, M3, and M4), T315I-ABL001 (M1′, M2′, and M3′) and T315I-ABL001-NIL (M1″ and M2″), with the A-loop colored in orange and αC-helix in pink.

Based on the results above, T315I-NIL, T315I-ABL001 and T315I-ABL001-NIL systems were selected to perform PCA to interrogate the overall free energy landscapes of the three relevant systems. The two most dominant collective principal components (PC1 and PC2) were used to project the overall conformation ensembles onto two dimensions (2D) plots to elaborate the conformational dynamics of the BCR-ABL1 during simulations. As shown in Figure 6A, T315I-NIL system exhibited four dominant conformations. The conformational ensembles of BCR-ABL1 were significantly changed upon ABL001 binding regardless of the presence or absence of NIL (Figures 6B,C). The T315I-ABL001 system had three major conformational clusters, whose global basin slightly shifted along the positive X-axis compared to the T315I-NIL system. The T315I-ABL001-NIL ternary complex, which contained two major structural clusters, had the lowest overall PCA values compared to the other two systems, indicating that the system had the least volatility.

To investigate their key conformational states during simulation, we applied PyEMMA (see Materials and Methods) for our complex systems to construct MSMs for analyses, which is powerful to probe into the protein conformations, unraveling unprecedented dynamic details (Lu et al., 2019b). Our models were first confirmed to be Markovian by the implied time scale tests (Supplementary Figure S2) and the Chapman-Kolmogorov tests (Supplementary Figure S3). Conformational ensembles for T315I-NIL, T315I-ABL001 and T315I-ABL001-NIL were then clustered into 4 (M1, M2, M3, and M4), 3 (M1’, M2’ and M3’) and 2 (M1” and M2”) MSM metastable states, respectively (Figures 6A–C). Comparative analysis of these dominant conformations showed that the whole backbone structure of BCR-ABL1 was stable during simulations, regardless of whether the kinase was successfully inhibited by drug.

The conformations of M1, M2, M3 and M4 in the T315I-NIL system all exhibited an A-loop open conformation and the αC-helix of M1, M2 and M4 showed a in conformation, implying their activated statuses. In the T315I-ABL001 system, the A-loop and αC-helix of M1’ are in an intermediate state, and the A-loop of M2’ exhibited an open state but αC-helix showed a distinct out conformation, implying that M1’ and M2’ were in an intermediate state. In contrast, the dominant conformation of M2’, which accounted for about 54.6% of the structural cluster, showed an A-loop closed and αC-helix out conformation, revealing that ABL001 allosterically regulated the global conformation of T315I-ABL001 complex shifted towards an inactive state. Significantly, in the T315I-ABL001-NIL system, the dominant conformation of M2”, which accounted for 75.5% of the overall structural ensemble, exhibited an inactive A-loop closed and αC-helix out conformation. The highest proportion of inactive conformation in the ensemble of the ternary complex system suggested that the co-binding of NIL and ABL001 might be the most effective in BCR-ABL1 inhibition, highlighting the synergistic effects between them.

Overall, conformational landscape analyses through MSMs unraveled that the combinatory regimen of NIL and ABL001 might function through trapping BCR-ABL1 in an inactive topology, suppressing its kinase activity.

### Energetic Insights for Overcoming Drug Resistance

To investigate in-depth detailed mechanism by which ABL001 restores the action of NIL, the binding free energy (ΔG_binding_) between NIL and T315I BCR-ABL1 upon ABL001 binding or not was calculated using molecular mechanics/Poisson-Boltzmann surface area (MM/PBSA) methods. As shown in Table 1, the ΔG_binding_ of NIL to BCR-ABL1 kinase in T315I-NIL system was -9.12 ± 1.42 kcal/mol, while for the T315I-ABL001-NILsystem, the result was -13.11 ± 1.12 kcal/mol. For the ternary complex, the ΔG_binding_ was lower, implying the favorable binding of NIL and its higher affinity in this system, which could be the explanation for co-administration to overcome resistance in T315I mutation. Hence, dual-targeting overcomes drug resistance not only by stabilizing the dynamic conformation of BCR-ABL1, but also by enhancing the binding of NIL to kinase through the induction of ABL001.

**TABLE 1:** Binding free energy analysis (kcal/mol) for the interactions between NIL and T315I BCR-ABL1 in T315I-NIL system and T315I-ABL001-NILsystem^a^.

Energy component (kcal/mol)	T315I-NIL	T315I-ABL001-NIL
ΔG_gas_	−101.34 (1.10)	−102.82 (1.39)
ΔG _solv_	92.22 (1.14)	89.71 (1.44)
ΔG_binding_	−9.12 (1.42)	−13.11 (1.12)

a Numbers in the parentheses are the standard deviations.

**FIGURE 7:**
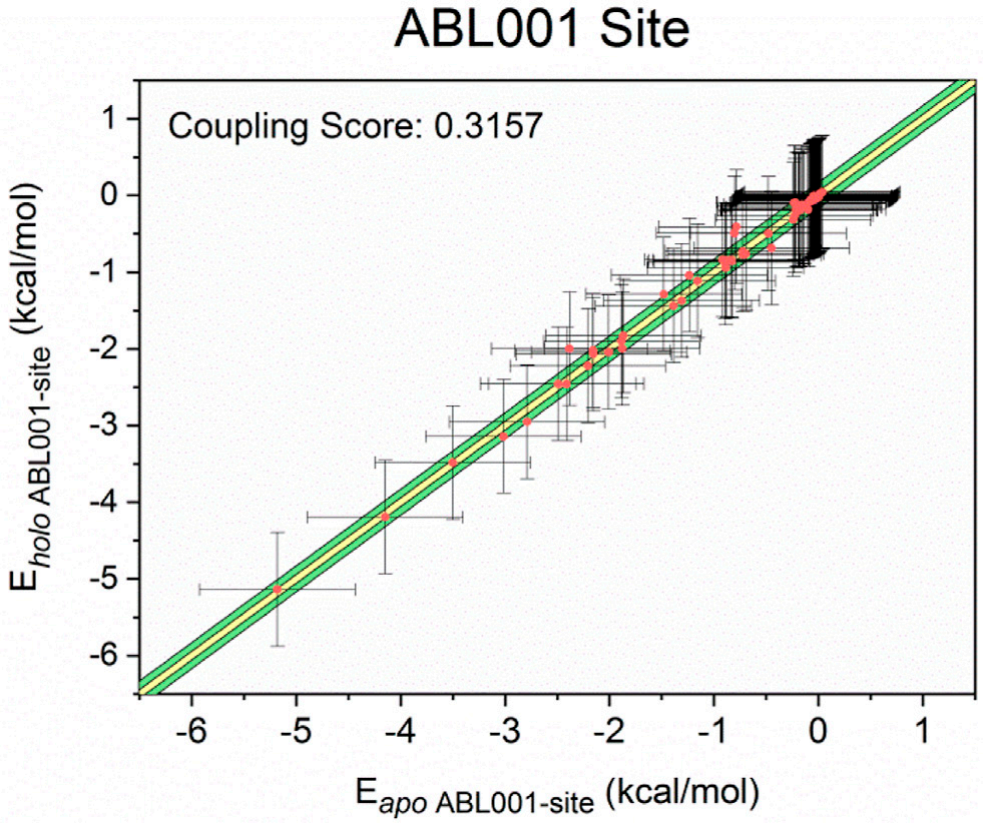
Energy coupling analysis for the ABL001 site in *apo- (unbound)* and *holo- (NIL-bound)* BCR-ABL1. Each dot stands for a residue pair, and its intersection indicates the corresponding standard deviation. The yellow region indicated minor energy differences (one standard deviation, group a), the green regions indicated moderate energy differences (three standard deviations, group b), and the area beyond the green area represented major energy differences (greater than three standard deviations, group c).

Finally, for more insight into the synergistic effect of NIL and ABL001 to inhibit BCR-ABL1, a computational scheme was established to confirm the coupling between the orthosteric and allosteric sites (Ma et al., 2016). Previous studies have modeled residue-residue interactions in which a number of residue pairs within the allosteric sites displayed massive interaction energy alters upon ligand binding (Ni et al., 2020b; Zhang et al., 2021). On the basis of this model, we yielded a quantitative model that all residue pairs within the allosteric pocket were classified into three groups based on the difference in interaction energy before and after orthosteric binding: minor energy difference a), moderate energy difference b) and major energy difference c). The energy differences between residue pairs in the minor energy difference a) group were within one standard deviation of the mean (in the yellow area), while those in the moderate energy difference b) group were within three standard deviations (in the green area). In the major energy difference group c), the energy differences between residue pairs are distributed at least three standard deviations beyond the mean interaction energy change (outside the green area). We calculated the ratio of the number of residue pairs in group c) to the number of residue pairs in groups b) and c) as the energy coupling fraction, which represents the coupling between the orthosteric and allosteric sites. The energy coupling fraction threshold was 0.25, which was chosen based on a previous study. The energy coupling score for the allosteric ABL001 site is 0.3157 (Figure 7), which surpassed the threshold of 0.25. The residue-residue interaction free energy of a portion of the residue pairs within the ABL001 site changed considerably before and after NIL loading, suggesting that the orthosteric drug NIL perturbation leads to a reversal allosteric communication. The energy coupling fraction deciphered the crosstalk between orthosteric perturbations and allosteric pockets in an energetic perspective, reflecting the synergistic effect of orthosteric NIL and allosteric ABL001.

## Discussion

In the era of modern medicine, drug resistance is one of the leading challenges, posing great threats towards effective therapeutics, especially in infections and cancers (Beyer et al., 2018; Li et al., 2021). The development of new therapies to circumvent such conundrum are therefore urgently needed. Accumulating evidence suggests that allosteric drugs can target structurally diverse allosteric sites of the resistant orthosteric target, resensitizing the resistant target and thus restoring the efficacy of the orthosteric drug (Guarnera and Berezovsky, 2020). In addition, dual-targeting at allosteric and orthosteric sites could improve pharmacological effectiveness and cover a broader therapeutic spectrum than monotherapy. Hence, combinatory treatments are emerging as a novel trend, representing a revolutionary strategy to tackle drug resistance (Qiu et al., 2021b).

One of the quintessential examples of harnessing both orthosteric and allosteric drugs to overcome drug resistance is dual-targeting BCR-ABL1, one of the most crucial anti-cancer targets within tyrosine kinase family, for CML treatments. The co-administration of allosteric drug ABL001 and classical TKIs targeting BCR-ABL1, (including imatinib, NIL, and dasatinib) for the therapy of CML achieved persistent and complete regression of the malignancy and addressed historically notorious resistance. Importantly, a recent clinical trial (Clinical Trial Number: NCT02081378) confirmed the efficacy of ABL001 in combination with TKIs for the treatment of CML. Such regimen was later approved for marketing by the FDA, highlighting its great therapeutic potential. However, the specific conformational landscapes mechanism of combination drug use to overcome T315I resistance mutation is unclear and needs to be studied in depth.

Here, based on computational biology system method, we illustrated the mechanism of dual-targeting therapy with BCR-ABL1. To gain an atomic structural view, a comparative MD simulation study and relevant analysis were conducted in the WT, WT-NIL, T315I, T315I-NIL, T315I-ABL001 and T315I-ABL001-NIL BCR-ABL1 systems for in-depth investigations on drug combinations to overcome T135I resistance. RMSD and RMSF analyses unveiled that the T3I5I-ABL001-NIL system had the lowest deviation and fluctuation values, indicating the dual-targeting system had the most stable conformational dynamics. DCCMs were carried out to reveal correlated motions in BCR-ABL1. The residue interactions within T315I-ABL001-NIL system were the most stable, suggesting that the drug combination limited the correlated motions between structural domains. Furthermore, The visualized community network clearly depicted the path and intensity of the allosteric crosstalk in each system, unveiling double targeting at allosteric and orthosteric sites could reduce the signal flow within BCR-ABL1. Conformational landscapes were also analyzed by MSM. The dominant MSM metastable state of T315I-ABL001-NIL system elucidated that the overall conformation of T315I-ABL001-NIL complex is preferred to be inactive compared to the conformation of T315I-NIL which tends to be in the activated state. Energetic analysis showed that the binding free energy between NIL and BCR-ABL1 was lower in the T315I-ABL001-NIL system, compared with T315I-NIL system, indicating the favorable binding of NIL and its higher affinity in the ternary complex. In conclusion, allo-loading could shift NIL-bound active form of BCR-ABL1 to a more stable inactive conformation by modulating the conformation of key structural domains such as A-loop and αC-helix through allosteric communication and induce stronger binding of NIL to BCR-ABL1, thus exerting concerted effects on improving the overall therapeutic efficacy.

Hence, our research revealed allosteric communication mechanism underlying dual-targeting at allosteric and orthosteric sites. The findings serve as proof of the concept for future clinical strategies to overcoming drug resistance that the appropriate combination of orthosteric and allosteric inhibitors could resolve drug-resistance as well as synergistically improve the efficacy of both drugs. According to our study, ABL001 could re-sensitize resistant TKI drugs by allosterically modulating the conformation of A-loop and C-helix. In the future, we can develop drug molecules to induce such conformational changes to restore recalcitrant drug-resistant kinases. And based on the identified allosteric pathways, it may be possible that drug allosteric potency could be enhanced by enhancing internal signal transduction within the C-lobe or SH2 or by strengthening he connection between Community two and the A-loop. And resistance residues outside the drug pocket could be predicted as mutations on the allosteric pathway may lead to resistance to allosteric drugs (Lu et al., 2019a, 2020). Moreover, based on reversed allosteric communication theory, we validated the coupling between the ATP-binding pocket and the allosteric site by energetic dynamics calculations. The residue-residue interaction free energy of a portion of the residue pairs within the ABL001 site changed considerably before and after NIL loading, suggesting that the orthosteric drug NIL perturbation leads to a reversal allosteric communication. The results supported the intrinsic linkage between topologically distinct sites, providing theoretical basis for future approaches using orthosteric modulators binding to predict allosteric pockets (Fan et al., 2021). Future research is expected to provide a more comprehensive insight into orthosteric ligands to fine-tune BCR-ABL1 and enable the mining of allosteric pockets with superior affinity and potency (Ni et al., 2021). The ABL001 pocket was also detected in Cyclin-dependent kinase 2 (CDK2), thus the investigation of BCR-ABL1 dual-targeting mechanism is useful to guide combination therapies with other kinases.

## Materials and Methods

### Construction of Stimulation Systems

In this study, six systems (WT, WT-NIL, T315I, T315I-NIL, T315I-ABL001 and T315I-ABL001-NIL) were constructed. The initial structure for T315I BCR-ABL in complex with NIL and NIL was obtained from Protein Data Bank (PDB ID: 5MO4) (Wylie et al., 2017). The missing residues (Thr277, Met278, and Gly383-Lys400) in the original crystal profile were modeled using available X-ray structures of relevant homologs (PDB ID: 3XOZ) by Discover Studio 3.1. Based on the T315-ABL001-NIL structure, the structures of T315I-ABL001, T315I-NIL and T315I were extracted from the T315I-ABL001-NIL complex. The I315 was mutated back to T in the WT and WT-NIL systems using Discovery Studio.

### MD Simulations Settings

MD simulations were performed for the six systems. We prepared the initial parameter files for minimizations and simulations utilizing Amber ff14SB force field (Maier et al., 2015) and general amber force field (GAFF) (Wang et al., 2004). A transferable intermolecular potential three-point (TIP3P) truncated octahedral water box (10 Å) was employed for solvation, and then the counterions were added for neutralization (Jorgensen et al., 1983). Subsequently, 0.15 mol L^−1^ NaCl was added into each system to attain the physiological conditions for proteins.

After the preparation, each system underwent two rounds of energy minimization with the steepest descent and conjugate gradient algorithm. Next, every system was heated from 0 to 300 K in 300 ps in a canonical ensemble (NVT), with an equilibrium runs of 700 ps. Finally, three independent rounds of 3 μs conventional MD simulations were conducted with random velocities for all systems under isothermal isobaric ensemble (NPT) condition and periodic boundary condition. During the MD simulation, the Particle Mesh Ewald (PME) method was performed to model the long-range electrostatic interactions (Darden et al., 1993), while a cutoff of 10 Å was set for the short-range electrostatic interactions and van der Waals force calculations. Furthermore, the SHAKE algorithm was performed to constrain covalent bonds involving hydrogens (Ryckaert et al., 1977).

### Dynamic Cross-Correlation Matrix Analysis

Using the CPPTRAJ plugin, the DCCM of all protein Cα atoms, which represents the fluctuations in Cα atom coordinates, was calculated to reflect the inter-residue correlations in each system (Hünenberger et al., 1995). The cross-correlation coefficient Cij was calculated according to the following Eq. 1:

$$C\left(i,j\right)=\frac{c\left(i,j\right)}{c{\left(i,i\right)}^{1/2}c{\left(j,j\right)}^{1/2}}$$ (1)

where *i* and *j* represent the *i*th and *j*th Cα atoms, respectively.

### Community Network Analysis

Utilizing the NetworkView plugin in VMD (Eargle and Luthey-Schulten, 2012), the community organizations of each system were calculated based on the correlation coefficient matrix C_ij_. The whole ABL1 of each system was recognized as a group of nodes (assigned to the Cα atom of each residue) connected by edges, which were drawn between nodes that remained within a cut-off distance of 4.5 Å for at least 75% of the simulation process (Sethi et al., 2009). We calculated the edge connections between certain nodes by Eq. 2:

$$d_{i,j}=-\log\left(\left|C_{i,j}\right|\right)$$ (2)

where i and j represent two nodes and Cij was calculated by Eq. 1.

Then, optimal pathways between all pairs of nodes were calculated with Floyd–Warshall algorithm. The gncommunities program was employed to get the substructure of the communities, which embedded the Girvan-Newman divisive algorithm and applied edge betweenness defined as the number of paired optimal paths. To acquire the optimal substructure of the network, edges with the highest betweenness would be iteratively removed from the network, and the remaining edges would be recomputed until each node represents an isolated community. Communities with residues less than three were discarded. Connectivity between communities was measured by the betweenness value.

### Energy Coupling Score Calculation

The molecular mechanisms generalized Born surface area (MM-GBSA) energy decomposition calculation was applied for the allosteric pockets on ABL^T315I^ in both NIL-bound (apo) and NIL-unbound (holo) systems to compare the residue-residue interactions, based on MD simulation trajectories. The interaction free energy for residues pairs separated by at least three amino acids in the sequence of the cavity was calculated by Eq. 3:

$$E=E_{\mathrm{int}}+E_{eel}+E_{vdw}+G_{pol}+G_{sas}$$ (3)

where E_int_ denotes internal energy, E_eel_ indicates electrostatic energy, E_vdw_ indicates van der Waals energy, G_pol_ represents the polar solvation free energy, and G_sas_ is the solvent accessible surface energy.

The energy coupling scores were assessed using the energy differences between the allosteric pockets in the holo and apo systems.

## Construction of Markov State Model

Based on the coordinates of T315I-NIL and T315I-ABL001-NIL, the PCA of overall protein backbone during the simulation of the two systems was calculated. and then taken as input for PyEMMA MSM analysis. The Python library PyEMMA (http://www.emma-project.org/latest/) was employed to estimate and validate Markov state models (MSM) based on MD simulation data (Scherer et al., 2015). By implied timescale validation, we confirmed that the T315I-ABL001-NIL and T315I-NIL systems were Markovian (Supplementary Figure S1) and reliable with a 100 microstate model with a lag time of 60 ns and a maximum k-means iteration number of 100. Next, based on the Perron cluster analysis (PCCA+) algorithm, the microstates were clustered into three macrostates in T315I-ABL001-NIL system and four macrostates in T315I-NIL system, respectively, which was further validated by the Chapman–Kolmogorov test (Supplementary Figure S2) (Prinz et al., 2011).

In each transferable state, we extracted trajectories that include more than 50% snapshots of the corresponding state using the “coordinates.save_traj” algorithm. The mdtraj package was used to extract the structures near the microstate cluster centers of each macrostate into the trajectories of corresponding macrostates. The representative conformation of each macrostate was selected based on the similarity score S_ij_.

$$S_{ij}=e^{-d_{ij}/d_{scale}}$$ (4)

In Eq. 4, the structure with the highest S_ij_ among the trajectories was considered as the most representative conformation of the macrostate. The d_ij_ represents the RMSD between the conformations i and j, while dscale is the standard deviation of d.

## Data Availability

The original contributions presented in the study are included in the article/Supplementary Material, further inquiries can be directed to the corresponding authors.
