# Supplementary material for: Mechanistic Insights Into Co-Administration of Allosteric and Orthosteric Drugs to Overcome Drug-Resistance in T315I BCR-ABL1
Source: Front Pharmacol. 2022 Mar 18;13:862504. doi: 10.3389/fphar.2022.862504 (PMC8971931; doi:10.3389/fphar.2022.862504)
Supplement: Supplementary file 1 [file DataSheet1.docx]

**Supplemental Information**

**Mechanistic insights into co-administration of allosteric and orthosteric drugs to overcome drug-resistance in T315I BCR-ABL1**

**Hao Zhang^1,2,#^, Mingsheng Zhu^3,#^, Mingzi Li^1,#^, Duan Ni^4^, Yuanhao Wang^4^, Liping Deng^1^, Kui Du^1,*^ Shaoyong Lu^4,*^, Hui Shi^5,*^, Chen Cai^4,*^**

^1^School of Chemistry and Chemical Engineering, Shaoxing University, Shaoxing 312000, China

^2^Department of Plastic and Reconstructive Surgery, Shanghai Ninth People's Hospital, Shanghai Jiao Tong University, School of Medicine, Shanghai, 200011, China

^3^Department of Anesthesiology, Huashan Hospital Affiliated to Fudan University, Shanghai 201907, China

^4^Medicinal Chemistry and Bioinformatics Center, Shanghai Jiao Tong University, School of Medicine, Shanghai 200025, China

^5^Department of VIP Clinic, Changhai Hospital, Navy Medical University, Shanghai, 200433, China

^6^Department of Respiratory and Critical Care Medicine, Changhai Hospital, Navy Medical University, Shanghai, 200433, China

^#^ These authors contributed equally to this work.

*****Correspondence:

Dr. Shaoyong Lu; [lushaoyong@sjtu.edu.cn](mailto:lushaoyong@sjtu.edu.cn)

Dr. Kui Du; dkui@usx.edu.cn

Dr. Hui Shi; pooh_shi@163.com

Dr. Chen Cai; Cai_chen1978@163.com


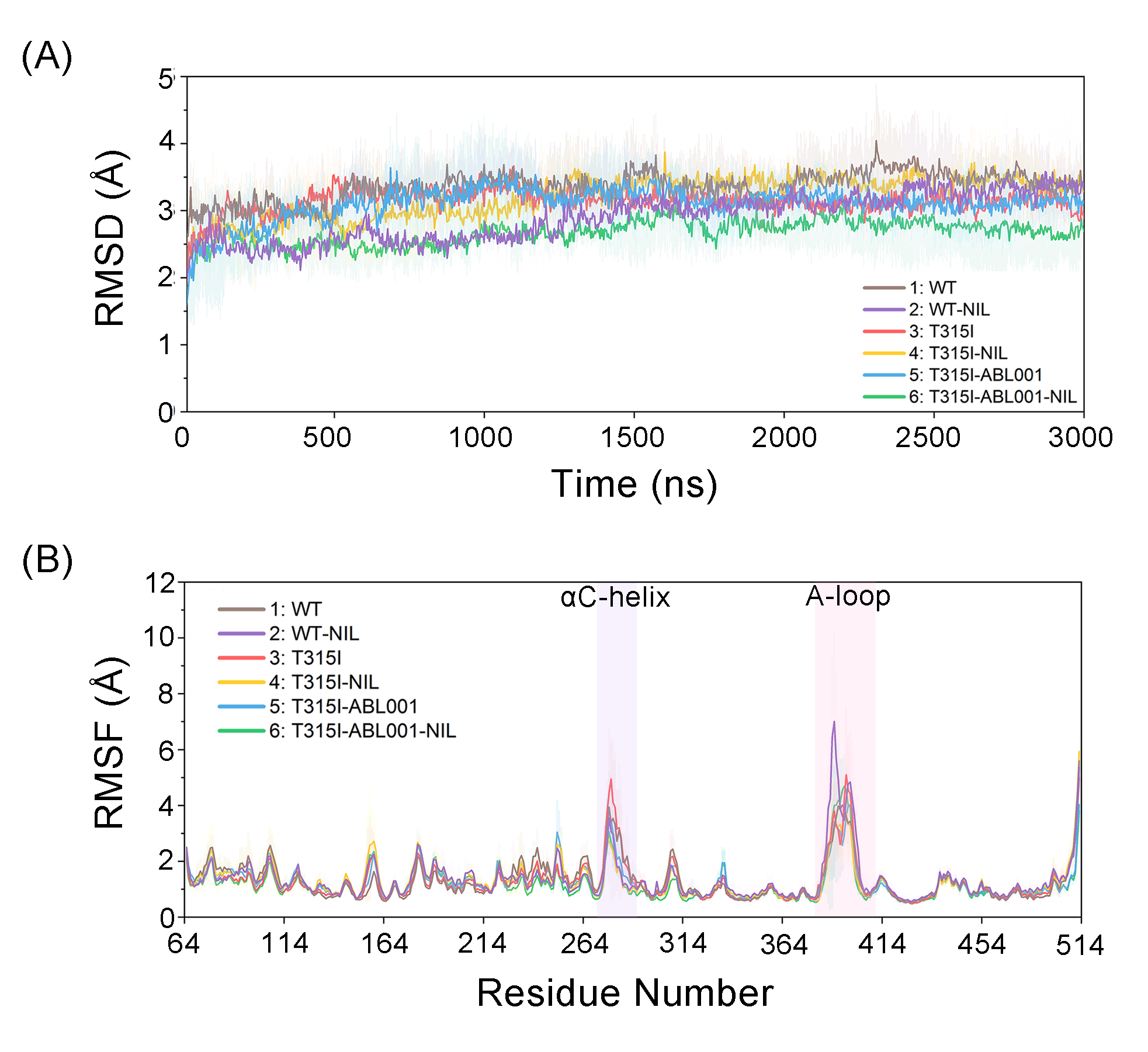


**Figure S1. (A)** The Cα RMSD of the protein for the WT (brown), WT-NIL (purple), T315I (red), T315I-NIL (yellow), T315I-ABL001 (blue) and T315I-ABL001-NIL (green) systems along 3000 ns MD simulations. **(B)** The Cα RMSF of WT (brown), WT-NIL (purple), T315I (red), T315I-NIL (yellow), T315I-ABL001 (blue) and T315I-ABL001-NIL (green) systems along 3000 ns MD simulations (Residues 280-292 are αC-helix, and residues 381-402 are A-loop).


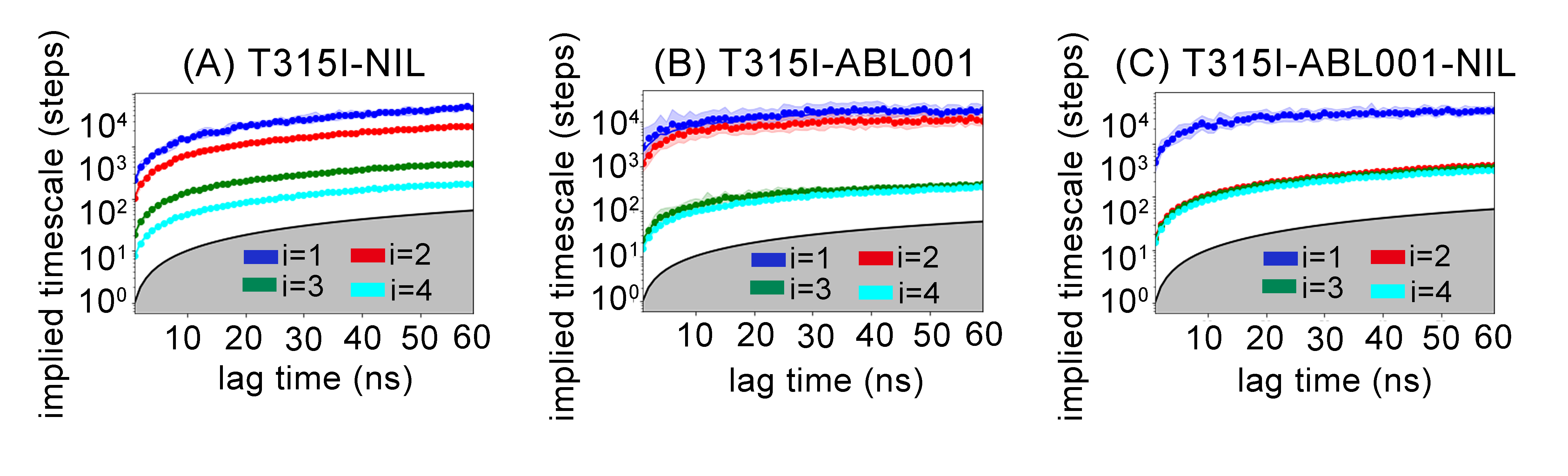


**Figure S2.** The result of implied timescale test for T315I-NIL **(A)**, T315I-ABL001 **(B)** and T315I-ABL001-NIL **(C)** complex. Blue, red, green, and cyan lines showed the timescale τ_1_, τ_2_, τ_3_ and τ_4_ as a function of lag times. Black line represents x=y in logarithmic coordinates.


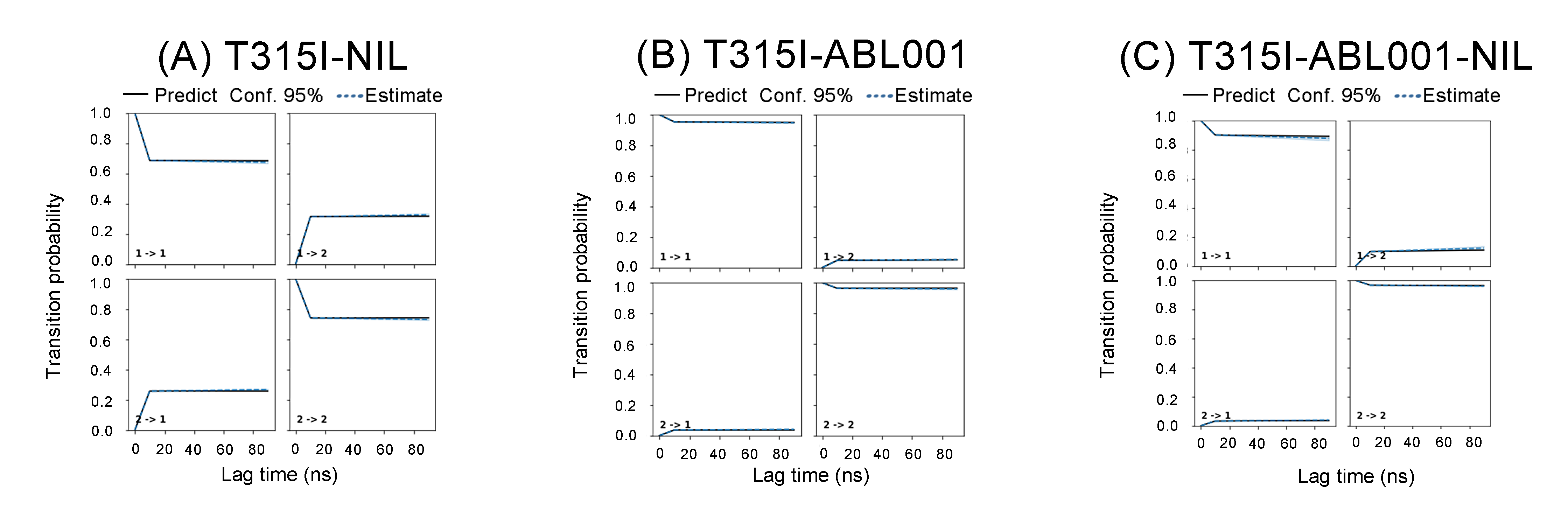
**Figure S3.** The results of the Chapman-Kolmogorov test of metastable states for T315I-NIL **(A)**, T315I-ABL001 **(B)** and T315I-ABL001-NIL **(C)** system.
